# Linguistic-Driven Partial Semantic Relevance Learning for Skeleton-Based Action Recognition

**DOI:** 10.3390/s24154860

**Published:** 2024-07-26

**Authors:** Qixiu Chen, Yingan Liu, Peng Huang, Jiani Huang

**Affiliations:** 1 College of Information Science and Technology, Nanjing Forestry University, Nanjing 210037, China; chenqixiu0618@njfu.edu.cn (Q.C.); hjiani@njfu.edu.cn (J.H.); 2 School of Computer Science and Engineering, Nanjing University of Science and Technology, Nanjing 210094, China; penghuang@njust.edu.cn

**Keywords:** skeleton-based action recognition, cross-modal, transformer

## Abstract

Skeleton-based action recognition, renowned for its computational efficiency and indifference to lighting variations, has become a focal point in the realm of motion analysis. However, most current methods typically only extract global skeleton features, overlooking the potential semantic relationships among various partial limb motions. For instance, the subtle differences between actions such as “brush teeth” and “brush hair” are mainly distinguished by specific elements. Although combining limb movements provides a more holistic representation of an action, relying solely on skeleton points proves inadequate for capturing these nuances. Therefore, integrating detailed linguistic descriptions into the learning process of skeleton features is essential. This motivates us to explore integrating fine-grained language descriptions into the learning process of skeleton features to capture more discriminative skeleton behavior representations. To this end, we introduce a new Linguistic-Driven Partial Semantic Relevance Learning framework (LPSR) in this work. While using state-of-the-art large language models to generate linguistic descriptions of local limb motions and further constrain the learning of local motions, we also aggregate global skeleton point representations and textual representations (which generated from an LLM) to obtain a more generalized cross-modal behavioral representation. On this basis, we propose a cyclic attentional interaction module to model the implicit correlations between partial limb motions. Numerous ablation experiments demonstrate the effectiveness of the method proposed in this paper, and our method also obtains state-of-the-art results.

## 1. Introduction

Action recognition [1,2,3,4,5] constitutes a pivotal branch within the computer vision field, dedicated to identifying human or object behaviors and actions through the analysis of visual information contained in video sequences or real-time video streams. This technology plays a crucial role in diverse applications such as human–computer interaction [6,7,8,9,10], health rehabilitation [11,12,13], and sports analysis [14,15,16]. The advent of depth sensors, exemplified by the Kinect [17], has facilitated easy access to human skeleton joint data. Currently, skeleton-based action recognition has garnered substantial interest for its computational efficiency and inherent robustness against variations in lighting conditions, viewpoints, and background noise.

**Figure 1:**
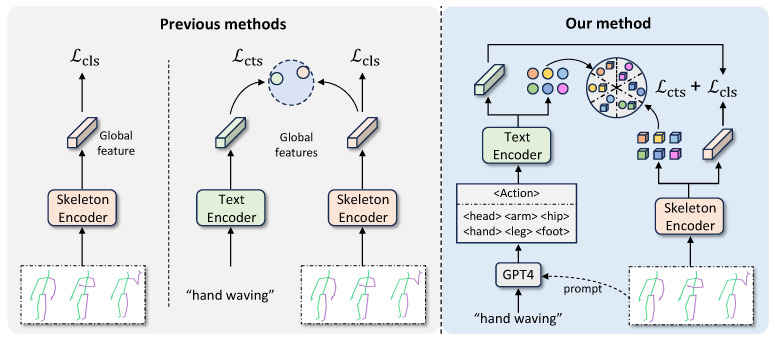
Idea of this work. Most previous methods (as shown above **left**) employ a single encoder to extract global features, or (as shown above **middle**) introduce the text information to conduct extra contrast loss. Nevertheless, there are instances where the visual semantic similarities or ambiguities between different actions make it challenging to distinguish between them. In contrast, as shown above **right**, we generate local feature descriptions of actions to learn finer-grained representations of skeleton limb motion. Meanwhile, the cyclic attention interaction module is proposed to mine the implicit association between partial limb motions.

Research on skeleton-based action recognition [18,19,20,21] from the perspective of network architecture can be broadly categorized into four types: methods based on Recurrent Neural Networks (RNNs) [22,23,24], methods based on Convolutional Neural Networks (CNNs) [15,25], methods based on Graph Neural Networks (GCNs) [26,27,28], and Transformer-based methods [9,19,29]. A frequently employed pipeline is to convert raw skeleton data into data formats associated with point sequences or graphical structures, subsequently applying the aforementioned deep learning techniques for feature extraction. RNN-based methods [30,31] recursively process data sequences, effectively capturing temporal dependencies, but may suffer from challenges with complex spatio-temporal data and long-term dependencies. CNN-based methods [18,32] perform convolutional operations within designated spatial or spatio-temporal windows to progressively extract higher-level features, exhibiting translation invariance. GCN-based methods [33,34,35,36] leverage the graph topology of the human skeleton to capture the relationships between different nodes. However, this approach is constrained in its ability to identify relationships between nodes that are not directly edge-connected (e.g., “head” and “feet”). Transformer-based methods [20,29] benefit from the self-attention mechanism, offering advantages in modeling long-distance dependencies and unrelated nodes, and have gradually become one of the most popular research frameworks in the community. Consequently, this work aims to explore a more effective skeleton activity representation based on Transformer (Figure 1).

To enhance the skeleton-based activity representation, researchers often introduce additional modalities, such as video (RGB) and depth image sequences [37,38,39], as supplementary information. Nevertheless, the additional processing and computation of modal data, as previously described, will result in extra computational overheads. Therefore, we expect to discuss a balanced learning strategy between performance and cost to effectively represent skeleton activity. Xiang et al. [21] proposed a cross-modal skeleton activity recognition method called Generative Action-description Prompts (GAP), which introduces a pre-trained large language model to generate textual descriptions of body parts’ actions and serves as supervised information to constrain the optimization of different body parts in the skeleton modality. On the one hand, GAP prompts further reflection on the role of textual descriptions in skeleton-based action recognition. There are visual semantic similarities among different body actions; for instance, “side kick” and “kicking” both involve leg movements, but skeleton data alone fails to effectively capture the nuanced motion patterns of these fine-grained behaviors [4]. Language, however, could provide a more nuanced and discerning form of guidance. On the other hand, there is implicit synergy among local body movements when a specific action occurs. For instance, there are simultaneous spatio-temporal displacements of the “head” and “hands” during the action “sneeze”. Consequently, how to sufficiently mine the semantic associations among these local body movements poses a significant challenge.

To alleviate the above two problems, we propose a fine-grained cross-modal skeleton action recognition approach, namely Linguistic-Driven Partial Semantic Relevance Learning (LPSR), which consists of two major components: the Partial Semantic Consistency Constraints (PSCC) and the Cyclic Attention Interaction Module (CAIM). In PSCC, we leverage the current state-of-the-art large language model to generate more detailed local body movement descriptions, as well as the global description of the action, by using skeleton point visualizations and text labels as inputs. Multiple local body descriptions guide the model to learn finer-grained representations of skeleton body movements, where the Kullback–Leibler (KL) consistency loss is used to construct local semantic consistency associations across modalities. Global textual descriptions are then (as key and value) associated with the global skeleton feature to learn a more discriminative action feature via cross-attention. Furthermore, considering the semantic synergy between local body movements, we design the CAIM module to model the implicit relations between them. The local body parts studied in this paper include the “head”, “arm”, “hand”, “hip”, “leg”, and “foot”. The selection of these parts is mainly based on the division of the human body into 25 nodes based on the dataset. We locally segment the human body based on the information provided by these nodes. In summary, the main contributions of this paper are summarized as follows:

1. We propose a novel Linguistic-Driven Partial Semantic Relevance Learning framework (LPSR) for skeleton-based action recognition. The framework leverages the powerful zero-sample capability of multi-modal large language models to generate global and local textual descriptions of skeleton actions, and furthermore constructs cross-modal partial semantic consistency constraints to guide the model to learn a more discriminative representation;
2. We propose a Cyclic Attention Interaction Module (CAIM) to mine the implicit semantic associations between different body movements, fully exploiting the potential of synergistic relationships of local body movements in global action understanding.
3. We conduct extensive ablation studies on two popular benchmarks NTU-60 and NTU-120, and the experimental results demonstrate the effectiveness of the proposed method in this work. In addition, compared with previous Transformer-based methods, our method also achieves state-of-the-art results under the same setup conditions.

## 2. Related Works

**Skeleton-based Action Recognition.** Skeleton-based Action Recognition [35,40,41,42] is a technique for recognizing human movements by capturing and analyzing the movements of human skeleton joints. Human joint trajectories [27,43] offer a detailed perspective on human movement, largely due to the spatial information they encompass and their strong correlation with adjacent joint nodes. However, representing skeleton information has its challenges: it is often sparse and noisy. This sparsity becomes evident when distinguishing between similar actions, like ‘brushing teeth’ and ‘brushing hair’, which are almost identical in body movement and heavily rely on hand movements for accurate identification [4]. Recently, deep learning, propelled by advances in high-performance computing and technology, has shown remarkable capabilities in extracting complex features. One area where deep learning is particularly effective is in processing time-series data through Recurrent Neural Networks (RNNs) [44,45,46,47]. RNNs excel in learning dynamic dependencies within such data. However, they face limitations in modeling spatial dependencies among skeleton joints. To address this, Du et al. [24] proposed an innovative solution: an end-to-end hierarchical RNN framework. Complementing this approach, Yang et al. [48] introduced the concept of group sparse regularization. This technique centers on investigating the concurrent characteristics of skeleton joints, providing a more profound comprehension of their interrelations.

In addition to the RNN-based approach, Convolutional Neural Networks (CNNs) [25,32,43,49] are well-regarded for their excellent capability in extracting features and learning spatial dimensions, and have been successfully utilized to process spatio-temporal data in skeleton analyses. Wang et al. [43] and Li et al. [18] encode the skeleton sequence data into an image and then feed it into a CNN for action recognition, giving a skeleton spectrogram and a joint trajectory map, respectively. Wang et al. [50] converted skeleton joints into multiple 2D pseudo-images to suit the CNN’s input needs, enabling the network to capture spatio-temporal characteristics. Additionally, Xu et al. [49] introduced a solely CNN-based structure known as Topologyaware CNN, designed to enhance the modeling of irregular skeleton topologies by CNNs.

Yet, the aforementioned techniques struggle to grasp the inter-joint correlations, Yan et al. [26] depict the human body as a graph, characterizing joint connections with an adjacency matrix, and introduce the Spatio-Temporal Graph Convolutional Network (ST-GCN). This network addresses the temporal and spatial dimensions of the convolution and processes the skeleton data for efficient modeling. In addition, combining semantic information of human joints and frames [21,51] has been shown to enrich the expressiveness of skeleton features, thus improving recognition accuracy. Diverging from these graph-centric methods, our approach models skeleton data using Linguistic-Driven Semantic Relevance Learning, offering a distinctive outlook that could yield novel insights and advancements in the domain of action recognition and pose estimation.

**Transformer-based Action Recognition.** In recent years, there has been a notable shift in Natural Language Processing (NLP) [1,51,52] towards the adoption of Transformer structures [53] as a replacement for traditional network architectures. Due to the powerful long-range temporal modeling capabilities of Transformers with self-attention modules, there has been a growing interest in utilizing Transformers for action recognition tasks. While most existing approaches in this area utilize video frames as input tokens [54,55], a limited number of techniques integrate skeleton data [9,19] within the Transformer architecture. Nonetheless, the computational demands for Transformer-based action recognition are substantial, given the self-attention mechanism’s application to numerous 3D tokens in videos. Self-attention is becoming increasingly popular in computer vision and has been applied to a variety of tasks, including image classification and segmentation [56,57], object detection [58], and action recognition [20,52]. In video action recognition, ref. [52] used self-attention to learn spatio-temporal features from frame-level patch sequences. Ref. [20] uses self-attention in skeleton-based action recognition instead of regular graph convolution. In contrast, our approach solely relies on self-attention to model skeleton data and calculates the correlation of all joints across multiple consecutive frames simultaneously.

**Language Model in Skeleton-Based Action Recognition.** Significant progress has been made in advanced natural language processing systems based on deep learning techniques with the introduction of models such as Bidirectional Encoder Representations from Transformers (BERT) [59]. These models are pre-trained to understand and generate complex text [60,61,62,63], capturing linguistic nuances and deeper meanings. Despite its effectiveness, the application of BERT was initially constrained to single-task adaptations, which limited its efficiency. In response to this limitation, the concept of Prompt Learning (PL) was introduced. This technique [63,64] enhances the adaptability of pre-trained LLMs to multiple tasks by adding specific textual parameters to the model’s input.

The principles of PL and transformer-based learning have been extended to Skeleton-Based Action Recognition. A notable example is GAP [21], which uses the Contrastive Language–Image Pretraining (CLIP) training method for skeleton action recognition and incorporates an additional transformer layer that significantly improves bone-based action recognition. In this framework, a cue learning (PL) technique is employed to construct bone-to-text correspondences, i.e., textual cues are used to allow GPT-3 [61] to generate detailed descriptions for different skeleton action categories for multimodal representation learning. This advancement demonstrates the great potential of transformer-based modeling and PL techniques for enhancing human action understanding and recognition using skeleton data. In contrast, we use GPT-4 [60] as a knowledge engine to enhance the understanding of actions. Textual cues and intuitive motion dynamics diagrams are input to generate global descriptions of human motion and local descriptions of different limb motions in an action to further optimize local behavioral learning, thus improving the quality of the learned representations. In addition, we aggregate global skeleton point representations and textual representations to form a cross-modal behavioral representation with broader applicability.

## 3. Methods

In this section, we first introduce the general framework for Linguistic-Driven Partial Semantic Relevance Learning in the sub-section Overview. Then, we will elaborate on the Cyclic Attention Interaction Module (CAIM) and Partial Semantic Consistency Constraints (PSCC) in detail, respectively.

### 3.1. Overview

**Figure 2:**
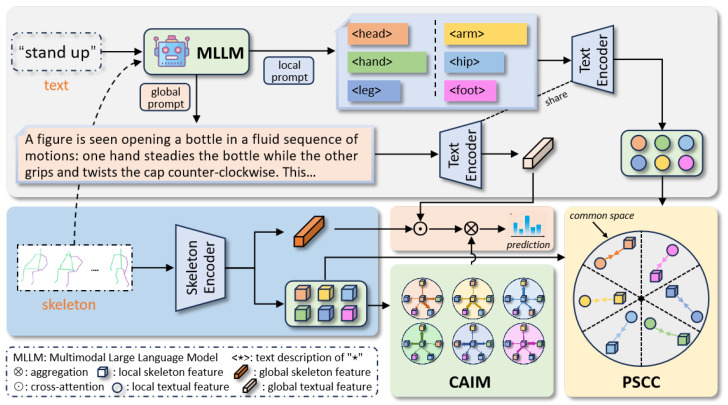
Overview of this approach. The novel framework is composed of two components: the Cyclic Attention Interaction Module (CAIM) and the Partial Semantic Consistency Constraints (PSCC). For a given raw skeleton input $X_{\mathrm{org}}$, we design a cyclic attention strategy to mine the potential relationships between partial limb motions in CAIM and output ${\tilde{f}}_{l}$. In PSCC, we use the text labels T as well as $X_{\mathrm{org}}$ as inputs to generate global and local descriptions $T_{g}$ and $T_{l}$; they are then encoded into common space to guide the learning of global action and local skeleton motions features using cross-modal aggregation and KL scatter alignment, respectively.

In this work, we propose a novel Linguistic-Driven Partial Semantic Relevance Learning framework for skeleton action recognition (shown in Figure 2), which contains two major sub-components: Cyclic Attention Interaction Module (CAIM) and Partial Semantic Consistency Constraints (PSCC).

For a given skeleton input $X_{\mathrm{org}}$, in CAIM, we first extract global skeleton features $S_{g}$ through a skeleton encoder and obtain local partial features $S_{l}$ based on node information. We design a cyclic attention strategy to mine the potential relationship between partial limb motions, and the output after local feature interaction is $f_{l}$. Each partial limb feature is then aggregated to obtain ${\tilde{f}}_{l}$. In PSCC, we use text labels T as well as $X_{\mathrm{org}}$ as inputs to generate global and local descriptions $T_{g}$ and $T_{l}$, and then obtain the encoded features $f_{g}$ and $f_{l}$, which can be passed through a pre-trained text encoder. We exploit these more discriminative textual descriptions to guide the learning of partial limb motion, specifically, using KL loss to construct local consistency constraints across modalities. In addition, we correlate global textual feature with global skeleton feature by cross-attention to obtain ${\hat{f}}_{g}$, which is fused with ${\tilde{f}}_{l}$ to obtain $f_{gl}$, using $f_{gl}$ to compute the classification objective. Finally, the final optimization objective is obtained $L_{total}$.

### 3.2. Cyclic Attention Interaction Module

Specifically, given the original skeleton sequence input $X_{\mathrm{org}}\in R^{C\times T\times V}$ with *T* frames and *V* joints. Following [19], we first expand the original skeleton sequence $X_{\mathrm{org}}$ to $X_{1}\in R^{C_{1}\times T\times V}$ in the channel dimension, by using a feature mapping layer (implemented by a Conv2d layer + a BatchNorm layer + a LeakyReLU layer). The expanded $X_{1}$ is then fed into a spatio-temporal tuple encoding layer after a sequence division operation, with the output $X\in R^{C_{1}\times T\times V_{1}}$. Next, the global skeleton feature $S_{g}$ is extracted by

(1) $$S_{g}=\Lambda \left(\Upsilon \left(X\right)+\mathrm{PE}\right)$$

where PE is a sine and cosine positional embedding function, Λ(·) represents the ViT-based skeleton encoder, and Υ(·) is applied to convert *X* to Query, Key and Value as inputs of Λ.

As mentioned earlier, there is an implicit connection between different body parts during an action being performed. Therefore, further exploration of the potential relationships between these local movements of body parts may contribute to a better understanding of skeleton action representations. To this end, we first utilize node information to refine the global feature $S_{g}$ to *K* local partial features, which can be formulated as

(2) $$S_{l}=\mathrm{RF}\left(S_{g},Info_{K}\right)$$

where $Info_{K}$ denotes the set of body parts, i.e., {head,hands,arms,hips,legs,feet}, RF(·) means the refined processing. Thus, the output $S_{l}=\left\{S_{l}^{1},S_{l}^{2},\cdots ,S_{l}^{K}\right\},K=6$ means the set of partial limb motion features, with each $S_{l}^{i}$ is $R^{C\times T\times V_{i}},i\in \left\{1,2,\cdots ,K\right\}$.

Furthermore, to mine the implicit synergies between partial limb motions, we design a cyclic attention strategy to learn the relation between each partial limb motion and others, shown as

(3) $$f_{l}=CycAttn\left(S_{l}^{i},\;S_{l}^{j\neq i},\;\eta \right),\left(i,j\right)\in \left[1,K\right]$$

where CycAttn(·) is a cyclic attention which is implemented by several cross-attention, with a cyclic mechanism that each limb motion is regarded as the query and others are key and value, and the parameter η means the number of attention layers. The specific process is shown in Algorithm 1. As a result, the interacted local features can be refined as $f_{l}=\left\{f_{l}^{1},f_{l}^{2},\cdots ,f_{l}^{K}\right\},K=6$.
**Algorithm 1: Cyclic Attention $\mathrm{CycAttn}(S_{l})$****Input:** Partial limb motion features $S_{l}=\left\{S_{l}^{1},S_{l}^{2},\cdots ,S_{l}^{K}\right\}$**for each** i∈[1,K] **do:**     1. Calculate: $S_{rest}=Concatenate\left(S_{l}-\left\{S_{l}^{i}\right\}\right)$;     2. Calculate Query, Key and Value: $S_{query}=W_{q}S_{l}^{i}$, $S_{key}=W_{k}S_{rest}$, $S_{value}=W_{v}S_{rest}$;     3. Calculate $f_{l}^{i}=CrossAttn\left(S_{query},S_{key},S_{value}\right)=SoftMax\left(\frac{S_{query}S_{key}^{T}}{\sqrt{d}}\right)S_{value}$;     4. i←i+1.**end**where Concatenate means splice partial limb motion features other than $S_{l}^{i}$, $W_{q}$, $W_{k}$,and $W_{v}$ denote the projection weights, *d* is the channel dimension of $S_{query}$.**Output:** Interacted local features $f_{l}=\left\{f_{l}^{1},f_{l}^{2},\cdots ,f_{l}^{K}\right\}$.

Each local feature captures the most relevant local motion than its own in (3); next, we aggregate these local skeleton features by

(4) $${\tilde{f}}_{l}=\frac{1}{K}\sum_{i=1}^{K}AvgPool\left(f_{l}^{i}{|}_{T,V}\right),\;i\in \left[1,K\right]$$

where AvgPool(·) is a fusion function to aggregate each partial limb feature in the temporal (*T*) and the joint (V) dimensions, and the ${\tilde{f}}_{l}$ will be involved in the calculation of the final classification loss.

### 3.3. Partial Semantic Consistency Constraints

Although existing large language models demonstrate impressive zero-shot generation capabilities, they are constrained to the generation and expansion of linguistic modalities, being unable to generate reasonably accurate captions for specific visual contents. Therefore, in this work, we introduce a multi-modal large model that utilizes dynamic visualization of skeleton data as the visual input. By designing specific linguistic prompts, we generate descriptions related to global action and partial limb motion, respectively. Specifically, for a given skeleton sequence input $X_{\mathrm{org}}$, we first convert it into a 3-dimensional array dynamic graph $X_{\mathrm{dg}}$ to show the intuitive motion process (refer to the visual input in Figure 2 for an intuitive understanding).

**Figure 3:**
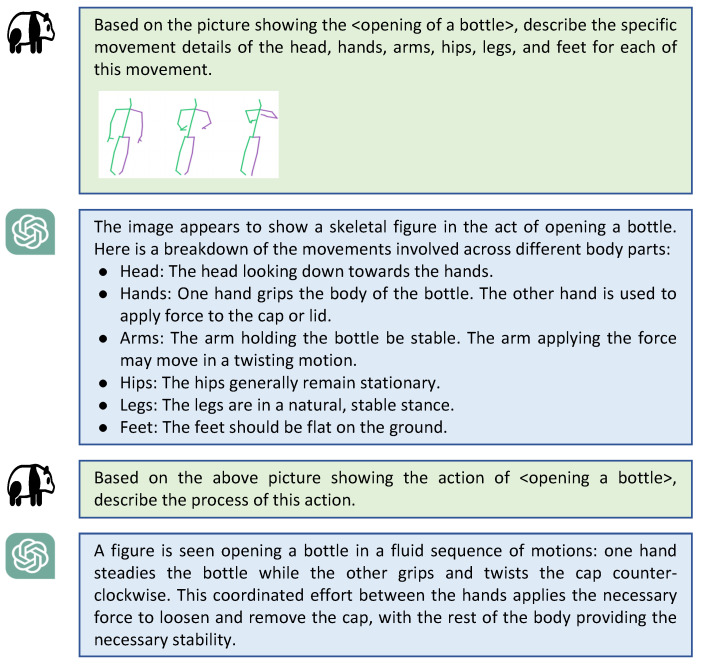
Textual action descriptions generated from two prompt input by GPT-4.

On this basis, we design two specific linguistic prompts targeting global actions and local limb motions, respectively, to generate more precise textual descriptions, which can be formulated as follows:

(5) $$T_{g}=G\left(X_{dg},T,P_{global}\right)$$

and

(6) $$T_{l}=G\left(X_{dg},T,P_{local}\right)$$

where G(·) indicates a multi-modal description generator which is implemented by GPT-4 in this work, making a groundbreaking advancement in understanding multi-modal information compared to previous versions. T denotes corresponding text labels, and $P_{global}$ and $P_{local}$ represent global action prompt and local limb motion prompt, respectively. The local output $T_{l}=\left\{\psi_{1},\psi_{2},\cdots ,\psi_{k}\right\}\;\left(k\in \left[1,6\right]\right)$ corresponds to the partial limb of body [“*head*”, “*arm*”, “*hand*”, “*hip*”, “*leg*”, “*foot*”]. The detailed content presentation of $T_{g}$, $T_{l}$, $P_{global}$ and $P_{local}$ are shown in Figure 3; the action category involved is exemplified by “openingabottle”.

The generated global and local detailed textual descriptions $T_{g}$ and $T_{l}$ are then encoded by

(7) $$f_{g}={\mathrm{LE}}_{g}\left(T_{g}\right),\;f_{l}={\mathrm{LE}}_{l}\left(T_{l}\right)$$

where ${\mathrm{LE}}_{g}\left(\cdot \right)$ and ${\mathrm{LE}}_{l}\left(\cdot \right)$ are frozen pre-trained language encoders that share parameters for each other, and the global and local textual features are $f_{g}$ and $f_{l}$, respectively.

Considering the similarities or ambiguities in the visual semantics between different actions, we introduce a partial semantic consistency strategy that utilizes the generated fine-grained local limb description as supervisory signals to guide the model in learning more discriminative representations of the partial limb motions:

(8) $$L_{cts}=\frac{1}{K}\sum_{i=1}^{K}KL\left(S_{l}^{i},\;f_{l}^{i},\;y_{l}^{i}\right)$$

where $L_{cts}$ represents the partial semantic consistency constraint, KL(·) is a standard KL contrast loss, $S_{l}^{i}$ and $f_{l}^{i}$ denote the *i*-th partial skeleton and textual features, respectively, and $y_{l}^{i}$ is the corresponding label for the KL function. We employ the KL divergence to align the cross-modal alignment for the partial limb motion.

### 3.4. Total Objective

In the CAIM and the PSCC modules, we discuss and explore the skeleton and language representation of the partial limb motions in detail. In addition, we also introduce the global language description to improve the comprehensiveness by

(9) $${\hat{f}}_{g}=CrossAttn\left(S_{g}W_{q},f_{g}W_{k},f_{g}W_{v},\delta \right)$$

where CrossAttn(·) indicates the cross-attention, $W_{q}$, $W_{k}$ and $W_{v}$ are the projection weights for the query, key, and value inputs, respectively, $S_{g}$ is defined as the query input, $T_{g}$ is denoted as the key and value inputs, respectively, and the δ means the number of layers for the CrossAttn.

Subsequently, the final representation is obtained by

(10) $$f_{gl}=Fus\left({\hat{f}}_{g},\;{\tilde{f}}_{l}\right)$$

where Fus(·) is an aggregation function to fusion the global and local feature, which can be implemented by a single MLP. The output $f_{gl}$ of (10) is then used to calculate the classification objective,

(11) $$L_{cls}=CEL\left(f_{gl},\;y\right)$$

where CEL(·) is a standard cross-entropy loss and *y* is corresponding action labels. Therefore, the final optimization objective of this work $L_{total}$ is the combination of $L_{cls}$ and $L_{cts}$ (obtained in (8)),

(12) $$L_{total}=\frac{1}{2}\left(L_{cts}+L_{cls}\right)$$

## 4. Experiments

In this section, extensive comparative experiments are conducted to demonstrate the effectiveness of our proposed method. The evaluation begins with a detailed description of the datasets utilized in our study. Following this, we outline the experimental setup. Subsequently, we conduct ablation studies using the NTU RGB+D skeleton data to determine the individual contributions of each component of our method. The final phase of our evaluation involves a comparison of the proposed method with existing state-of-the-art approaches, utilizing both NTU RGB+D 60 and NTU RGB+D 120 skeleton data sets.

### 4.1. Datasets

**NTU RGB+D 60.** The NTU RGB+D 60 dataset [65], a comprehensive resource for 3D human activity analysis, was developed and released by researchers at Nanyang Technological University, Singapore. This large-scale dataset comprises a diverse array of data types, including RGB, depth, infrared, and skeleton data. It encompasses 56,880 samples, representing a wide range of 60 human activity categories. The extensive size and varied nature of this dataset facilitate rigorous cross-subject (X-sub) and cross-view (X-View) evaluations, X-sub divides the dataset according to the person ID. The training set and the test set contain 20 subsets, respectively. X-View divides the dataset according to camera ID, substantially contributing to advancements in the field of 3D human activity analysis.

**NTU RGB+D 120.** The NTU RGB+D 120 dataset [66] represents an extension of the NTU RGB+D 60 dataset, encompassing all the data from the NTU RGB+D 60 and incorporating an additional 60 categories. This expansion results in a comprehensive collection of 120 categories, with a total of 57,600 newly added video samples, bringing the aggregate number of samples in the dataset to 114,480. It features high-resolution RGB videos at 1920 × 1080 pixels, while the depth maps and IR videos are captured at a resolution of 512 × 424. The 3D skeleton data includes the coordinates of 25 body joints per frame. For experimental assessment, the dataset offers two benchmarks: (1) cross-subject (X-sub) and (2) cross-setup (X-Set), catering to a wide range of research needs in the field. For X-Sub, the 106 subjects are split into training and testing groups. Each group consists of 53 subjects. The X-Set takes samples with even collection setup IDs as the training set and samples with odd setup IDs as the test set.

### 4.2. Experimental Setup

We follow the data processing procedure of [34] for NTU RGB+D 60 and NTU RGB+D 120. The skeleton encoder uses STTformer as the backbone network to extract the skeletal features and utilizes the Stochastic Gradient Descent (SGD) optimizer with a momentum of 0.9, a standard cross-entropy as the classification loss, weight decay of 0.0004, and batch size of 110. The learning rate is set to 0.1 initially and reduced by a factor of 10 at 60 and 80 epochs. For the text encoder, we load the pre-training weights of the text encoder to perform the inference process on the text descriptions (without training), and encode the text features. The temperature for contrastive loss is set to 0.1. Additionally, a warm-up strategy is applied during the first five epochs. We use PyTorch and all experiments are conducted on 2×Titan RTX 3090 GPUs. For a fair comparison, all settings are the same, except for the exploration subjects.

### 4.3. Ablation Study

In this section, We investigate the effectiveness of the proposed method through several experiments on the bone mode of the NTU-RGB+D 60 skeleton dataset.

**Ablation study for Cyclic Attention Interaction Module (CAIM).** To validate the potential synergy of limb motion, we design the CAIM module and perform ablation validation, and the results are recorded in Table 1. The notation “partial features (mean)” indicates that the global skeleton features are decoupled (to obtain head, hands, arms, hips, legs, feet) and then directly fusion with an average pooling layer, aggregating each partial limb feature in the temporal (*T*) and the joint (V) dimensions. The experimental results validate the effectiveness of our proposed module for CAIM. In contrast, the direct fusion of multiple partial limb features (mean) has limited performance improvement. Using CAIM to mine the synergy of each limb’s motion with other nodes has a positive impact on the action recognition of skeleton sequence.

**Ablation study for Partial Semantic Consistency Constraints (PSCC).** In order to verify the consistency constraints effect of local language descriptions on limb motion and the enhancement of global descriptions for the global skeleton representation, several ablation experiments are conducted. Firstly, the outcomes of the experiments utilizing partial and global descriptions, respectively, are present Table 2. The recognition of skeleton models without accompanying description information yielded the lowest accuracy. Following the introduction of partial descriptions, we observe a significant performance improvement, indicating that more detailed descriptive information about partial motion can effectively guide the model to learn more discriminative skeleton representations. Furthermore, the utilization of global descriptions also enhances the recognition performance. Notably, the optimal result is achieved by combining partial descriptions and global descriptions.

Furthermore, we assess the validity of different partial descriptions for the prediction, as shown in Table 3. The results obtained using a single local description are marginally higher than the baseline. The highest gain is achieved by using all six local partial descriptions corresponding to limb motions.

Finally, we demonstrate the ablations of distinct text encoders and record the results, as illustrated in Table 4. A comparison is conducted between four text encoders: BERT [59], DistilBERT [67], RoBERTa [68] and CLIP [63]. The results indicated that RoBERTa exhibited the most optimal performance. Given its commendable balance between efficiency and accuracy, RoBERTa was selected as the text encoder for this study.

**Ablation studies for different modules.** We perform distinct ablation studies on separate sub-components of the Cyclic Attention Interaction Module (CAIM) and Partial Semantic Consistency Constraints (PSCC) in prior experiments as a complement; this part provides ablation confirmation of the overall framework, as shown in Table 5. The integration of the CAIM module into the baseline model has been found to enhance its performance, indicating that cyclic attention interaction improves the model’s effectiveness. This improvement can be attributed to the CAIM module is capacity to effectively explore the implicit semantic relationships between different limb motions, thereby fully leveraging the synergistic potential of local limb motions within the global action context. Furthermore, the PSCC module improves performance by capitalizing on linguistic supervision and domain-specific knowledge of global action and local limb motions. This enables the model to learn more discriminative representations of skeleton action. The complete LPSR approach achieves optimal performance across both X-Sub and X-View. While each component of LPSR contributes differently to the overall performance, their combined effect significantly enhances the model’s accuracy when processing skeleton data.

**Figure 4:**
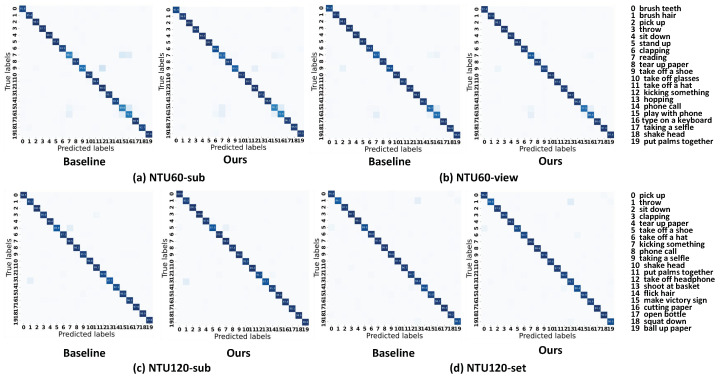
Confusion matrices for unimodal baseline and our methods.

**Visualization results.** In order to illustrate the efficacy of our methodology in a more visually compelling manner, we selected 20 action categories each from NTU60 and NTU120 to compare the baseline and our method using confusion matrices, as illustrated in Figure 4. In NTU60, actions such as “reading”,“taking off a shoe”,“playing with a phone”, and “typing on a keyboard” exhibited poorer classification performance. Our method significantly outperforms the baseline for these actions due to the text branch, which generates descriptions for different body parts involved in these actions. However, the performance of our proposed method for recognizing actions such as “tear up paper”, “phone call”, and “cutting paper” is degraded, probably due to the difficulty in recognizing objects in the skeleton. The generated text descriptions are mainly related to objects and local limbs, e.g., “paper cutting” and “paper tearing” both involve paper and hand descriptions, but due to the fine-grained nature of the skeletal data (small discriminative differences between actions), the final prediction results may be be guided by the text to favor incorrect categories with the same objects or localized limbs. Overall, our language-assisted action recognition method shows marked improvement.

### 4.4. Comparison with the State-of-the-Art Methods

We compared the performance of the LPSR method we developed with the current state-of-the-art methods on two datasets, NTU RGB+D 60 and NTU RGB+D 120. The results of the comparison of recognition accuracy are shown in Table 6. In our study, four different data integration strategies were used: bone, bone motion, joint, and joint motion. Meanwhile, we compared other state-of-the-art methods, including those based on LSTM, GCN and Transformer.

In comparing LSTM-based approaches, it is evident that our proposed LPSR framework shows a marked improvement over traditional LSTM-based models when applied to the dataset in question. The core limitation of LSTM-based methods lies in their struggle to effectively capture the spatial relationships between joints and bodily segments. On the other hand, GCN-based methods adeptly leverage the spatio-temporal characteristics of skeleton data, leading to superior recognition capabilities. When juxtaposed with a specific GCN-based approach, our LPSR methodology demonstrates distinct advantages, primarily due to the employment of linguistic supervision that steers the recognition of behavior. This supervision harnesses actionable insights from the interplay of movements and body parts, enriching the model’s representational power. Moreover, LPSR sets a new benchmark against Transformer-based counterparts. Ultimately, the consistent outperformance of LPSR across varied datasets underscores its efficacy and robustness as a state-of-the-art method in behavior recognition.

## 5. Conclusions

This study proposes a novel Linguistic-Driven Partial Semantic Relevance Learning framework (LPSR) for skeleton-based action recognition, which contains two major sub-modules: Cyclic Attention Interaction Module (CAIM) and Partial Semantic Consistency Constraints (PSCC). In comparison to previous methods, we introduce a more comprehensive multi-modal large-scale language model to generate more detailed linguistic descriptions of global actions and partial limb motions. Further, in PSCC, we generate multiple local body descriptions to guide the model to learn finer-grained representations of skeleton body motions. In addition, considering the semantic synergy between partial body motions, we propose the CAIM module to model the implicit relations between them. Extensive ablation experiments demonstrate the efficacy of the method present this paper, achieving comparable performance to the current state-of-the-art methods.

One limitation of our current approach to skeletal action recognition is its reliance on fully supervised conditions, which constrains its applicability in real-world scenarios where annotated data may be scarce. Future research will explore recognizing skeletal behaviors under weakly supervised or unsupervised conditions to broaden the practical utility of our methods. Another limitation is the small difference between the training and test set distributions in our skeletal action recognition task, which hampers the model’s performance when generalizing to new, unseen action classes. Consequently, enhancing the classification performance and generalization capabilities of our model in zero-shot skeletal behavior recognition will be a primary focus of our future work. 

## Figures and Tables

**Table 1:** Effect of CAIM evaluated on NTU RGB+D 60 Skeleton dataset in the bone mode. We record the recognition accuracy (%) for different settings X-Sub and X-View. Best results are in bold.

Methods	Accuracy (%)	
	X-Sub	X-View
Baseline	88.8	93.3
Baseline + partial features (mean)	88.9	93.3
Baseline + CAIM	**89.4**	**93.7**

**Table 2:** Influences of textual description types on the NTU RGB+D 60 Skeleton dataset in bone mode. We record the recognition accuracy (%) for different settings X-Sub and X-View. Best results are in bold.

Description Type	Accuracy (%)	
	X-Sub	X-View
Baseline	88.8	93.3
+ Partial Description	89.5	93.9
+ Global Description	89.1	93.5
+ Partial + Global Description	**89.6**	**94.1**

**Table 3:** Comparison of different body parts description on NTU RGB+D 60 Skeleton dataset in bone mode. We record the recognition accuracy (%) for different settings X-Sub and X-View. Best results are in bold.

Method	Text Partial Description						Accuracy (%)	
	Head	Hand	Arm	Hip	Leg	Foot	X-Sub	X-View
Baseline	✗	✗	✗	✗	✗	✗	88.8	93.3
	✓	✗	✗	✗	✗	✗	89.0	93.3
	✗	✓	✗	✗	✗	✗	88.9	93.5
	✗	✗	✓	✗	✗	✗	89.0	93.3
	✗	✗	✗	✓	✗	✗	89.1	93.4
	✗	✗	✗	✗	✓	✗	88.9	93.3
	✗	✗	✗	✗	✗	✓	88.8	93.3
	✓	✓	✓	✓	✓	✓	**89.5**	**93.9**

**Table 4:** Effect of text encoders evaluated on NTU RGB+D 60 Skeleton dataset in bone mode. We record the recognition accuracy (%) for different settings X-Sub and X-View. Best results are in bold.

Methods	Accuracy (%)	
	X-Sub	X-View
Baseline	88.8	93.3
BERT	89.1	93.8
DistilBERT	89.2	93.9
CLIP	89.5	94.0
RoBERTa	**89.6**	**94.1**

**Table 5:** Ablation studies for different modules on the NTU RGB+D 60 Skeleton dataset in bone mode. We record the recognition accuracy (%) for different settings X-Sub and X-View. Best results are in bold.

Methods	Accuracy (%)	
	X-Sub	X-View
Baseline	88.8	93.3
+ CAIM	89.3	93.7
+ PSCC	89.5	93.9
+ LPSR (CAIM + PSCC)	**89.8**	**94.2**

**Table 6:** Comparison of recognition accuracy with state-of-the-art methods on NTU RGB+D 60 and NTU RGB+D 120 Skeleton dataset. We record the NTU RGB+D 60 recognition accuracy (%) for different settings of X-Sub and X-View, and NTU RGB+D 120 recognition accuracy (%) for different settings of X-Sub and X-Set, respectively. Best results are in bold.

Type	Methods	NTU RGB+D 60		NTU RGB+D 120	
		X-Sub (%)	X-View (%)	X-Sub (%)	X-Set (%)
LSTM	ST-LSTM	83.0	87.3	63.0	66.6
	GCA	85.9	89.0	70.6	73.7
	2s-GCA	87.2	89.9	73.0	73.3
	LSTM-IRN	90.5	93.5	77.7	79.6
GCN	ST-GCN	81.5	88.3	78.9	76.1
	AS-GCN	89.3	93.0	82.9	83.7
	2s-AGCN	88.5	95.1	82.9	84.9
	MS-G3D	91.5	96.2	86.9	88.4
	CTR-GCN	92.4	96.8	88.9	90.6
	InfoGCN	92.7	**96.9**	**89.4**	90.7
Transformer	DSTA-Net	91.5	96.4	86.6	89.0
	IGFormer	**93.6**	96.5	85.4	86.5
	STTFormer	92.3	96.5	88.3	89.2
	LPSR	92.8	**96.9**	89.2	**90.8**

## Data Availability

Data are contained within the article.
